# The National Early Warning Score 2(NEWS2) to Predict Early Progression to Severe Community-Acquired Pneumonia

**DOI:** 10.3390/tropicalmed8020068

**Published:** 2023-01-17

**Authors:** Pattraporn Tajarernmuang, Pimchanok Sanwirat, Juthamas Inchai, Phichayut Phinyo, Atikun Limsukon

**Affiliations:** 1 Division of Pulmonary, Critical Care, and Allergy, Department of Internal Medicine, Faculty of Medicine, Chiang Mai University, Chiang Mai 50200, Thailand; 2 Department of Internal Medicine, Faculty of Medicine, Chiang Mai University, Chiang Mai 50200, Thailand; 3 Center for Clinical Epidemiology and Clinical Statistics, Faculty of Medicine, Chiang Mai University, Chiang Mai 50200, Thailand; 4 Department of Family Medicine, Faculty of Medicine, Chiang Mai University, Chiang Mai 50200, Thailand

**Keywords:** National Early Warning Score, community-acquired pneumonia, IDSA/ATS minor criteria, predictive performance, NEWS2

## Abstract

This study aimed to assess the predictive performance of the National Early Warning Score 2 (NEWS2) to identify the early progression to severe disease in patients with community-acquired pneumonia (CAP). A prospective-cohort study was conducted among patients with CAP admitted to a university hospital between October 2020 and December 2021. The endpoint of interest was the progression to severe CAP, defined as the requirement for a mechanical ventilator, a vasopressor, or death within 72 h after hospital admission. Among 260 patients, 53 (25.6%) had early progression to severe CAP. The median NEWS2 of the early progression group was higher than that of the non-progression group [8 (6–9) vs. 7 (5–8), *p* = 0.015, respectively]. The AUROC of NEWS2 to predict early progression to severe CAP was 0.61 (95% CI: 0.52–0.70), while IDSA/ATS minor criteria ≥ 3 had AUROC 0.56 (95% CI 0.48–0.65). The combination of NEWS2 ≥ 8, albumin level < 3 g/dL and BUN ≥ 30 mg/dL improved AUROC from 0.61 to 0.71 (*p* = 0.015). NEWS2 and IDSA/ATS minor criteria showed fair predictive-accuracy in predicting progression to severe CAP. The NEWS2 cut-off ≥ 8 in combination with low albumin and uremia improved predictive-accuracy, and could be easily used in general practice.

## 1. Introduction

Pneumonia is a major public-health problem in Thailand. In 2019, the Office of Epidemiology, Department of Disease Control, Ministry of Public Health Thailand found 254,876 pneumonia cases, representing an approximate rate of 384 cases per 100,000 population, with a mortality rate of 0.25 per 100,000 population. Two-thirds of pneumonia cases occurred in adults over 65 years and children under 5 years of age [1]. Community-acquired pneumonia (CAP) is caused by an infection that originates outside the hospital. The pathogens can be viruses, bacteria, and fungi, with a different prevalence in each age-group and immune status. The most common symptoms are fever, cough, shortness of breath, and chest pain. The symptoms of CAP can vary from mild symptoms to acute respiratory failure, depending on the type of pathogens, the severity of the disease, and the patient’s original health background. The diagnosis is based on clinical signs and symptoms, along with chest radiographs indicating lung parenchymal abnormalities [2]. Determining which patients have severe CAP is important, because studies have shown that patients directly admitted to the intensive care unit (ICU) had a statistically significantly lower mortality-rate than those who received delayed intensive treatment [3].

The CURB-65 score (confusion, uremia, respiratory rate, blood pressure, and age) is most commonly used to triage the site of care for patients with CAP; however, it seems to have favorable mortality-prediction but limited accuracy for identifying high-risk patients requiring intensive-care-unit (ICU) admission [4,5,6]. The official clinical-practice guidelines for diagnosis and treatment of adults with CAP 2019 approved by the American Thoracic Society (ATS) and Infectious Disease Society of America (IDSA) has recommended a level of care selection using the 2007 IDSA/ATS criteria. Patients who met one of two major criteria (the need for mechanical ventilation or vasopressors) or at least three of nine of the minor criteria were defined as having severe CAP and requiring intensive care [7]. Several validated studies of the 2007 IDSA/ATS criteria showed favorable predictive efficacy [8,9,10]. However, two systematic reviews and meta-analyses demonstrated the lower sensitivity of minor criteria in predicting the requirement for intensive care [5,6]. Moreover, because of the difficulty of using IDSA/ATS minor criteria in general practice with nine different parameters, several studies tried to simplify the score [11,12].

The National Early Warning Score (NEWS) was first published by the Royal College of Physicians (RCP) in 2012 [13] and updated to NEWS2 in 2017 [14], following improvements in the detection of, and response to, clinical deterioration in patients with acute illness. The NEWS2 assessment method focuses on simple data collection which is suitable for practical use. NEWS2 ≥ 7 indicates high clinical risk that requires an immediate response, usually with admission to the ICU. The recent study demonstrated the additional prognostic accuracy of the NEWS on the pneumonia severity index (PSI) and CURB-65 in predicting ICU admission [15].

To our knowledge, the comparative data between the NEWS2 and IDSA/ATS minor criteria has not been evaluated. Therefore, this study aimed to assess the predictive performance of NEWS2 in identifying CAP patients with early progression to severe disease, and compared it to the 2007 IDSA/ATS minor criteria.

## 2. Methods

### 2.1. Study Design

This was a prospective cohort study carried out at Chiang Mai University Hospital during October 2020 to December 2021. The study population was adult inpatients (age ≥ 18 years) diagnosed with CAP. The diagnosis of CAP required new or increasing lung-infiltration on a chest radiography, and at least one of the following symptoms or signs: fever, productive cough, dyspnea, chest pain, or new abnormal findings of the chest auscultation (crepitation or rhonchi). The exclusion criteria included patients with severe CAP who met at least one of two IDSA/ATS major criteria at admission, pregnant patients, and COVID-19 patients.

### 2.2. Data Collection

Data was collected using case record forms, with informed consent being taken at the first evaluation. The patients were followed from hospital admission until discharge. Evaluation of vital signs was carried out at the first visit to the emergency department (ED) by a nurse or suitably trained assistant nurse. Data pertinent to demographics, comorbidities, vital signs, symptoms and signs, laboratory, and imaging were obtained from the medical records. The decision to transfer to a general ward or ICU was left to the discretion of the treating physicians, who were not part of this study. Due to limited ICU resources, almost all patients who did not meet the major criteria were transferred to a general ward.

In all patients, NEWS2 was calculated as part of the routine at the Emergency Department. The NEWS2 includes six physiological assessments (respiratory rate, SpO_2_, systolic blood pressure, pulse rate, consciousness, and temperature) plus one weighting score (need for oxygen supplement), with each item having a detailed score. The total score indicates the severity of the acute illness [14].

The 2007 IDSA/ATS minor criteria were calculated on admission. The parameters included respiratory rate (RR) ≥ 30 breaths/min, PaO_2_/FiO_2_ ratio ≤ 250, multi-lobar infiltration, confusion or disorientation, uremia (blood-urea-nitrogen (BUN) level ≥ 20 mg/dL), leukopenia (white-blood-cell count < 4000 cells/µL), thrombocytopenia (platelet count < 100,000/µL), hypothermia (core temperature < 36 °C), and hypotension requiring aggressive fluid-resuscitation [7]. The CURB-65 score (confusion, uremia (blood-urea-nitrogen (BUN) level ≥ 20 mg/dL), RR ≥ 30 breaths/min, systolic blood pressure (SBP) < 90 mmHg or diastolic blood pressure (DBP) < 60 mmHg, and age ≥ 65 years) was also calculated on admission.

### 2.3. Endpoint of Interest

In general, most patients with CAP should achieve clinical improvement within 48–72 h after appropriate treatment [2]. The endpoint of interest was early progression to severe pneumonia, defined as the progression of the disease to respiratory failure requiring mechanical ventilation, circulatory failure requiring vasopressors, or death within 72 h after admission. Hospital mortality and hospital length-of-stay (LOS) were also evaluated.

### 2.4. Statistical Analysis

All statistical analysis was performed using STATA/MP version 14.2 (Stata Corp., College Station, TX, USA). Categorical data of baseline characteristics between early progression and non-progression groups were expressed as numbers and percentages, while continuous data were expressed using the median and interquartile range or mean and standard deviation. A comparison of categorical variables between groups was analyzed using the chi-square test or Fisher’s exact test. Continuous variables were analyzed, using Student’s *t*-test or the Wilcoxon rank-sum test, as appropriate for data distribution. A *p*-value < 0.05 was considered statistically significant. The predictive performance of NEWS2 and IDSA/ATS minor criteria for prediction of early progression to severe pneumonia was assessed, using the area under the receiver-operating-characteristic (AUROC) curves, with a 95% confidence interval (CI).

## 3. Results

### 3.1. Patient Population

A total of 53 (25.6%) of the 260 enrolled patients had early progression to severe pneumonia, in which 42 (79.2%) patients progressed to respiratory failure and required mechanical ventilation, 17 (32.1%) patients had hemodynamic instability requiring a vasopressor, and 6 (11.3%) patients died within 72 h. Overall demographic data such as gender, age, comorbidities, symptoms, signs, and the IDSA/ATS minor criteria presented on admission were not significantly different in both groups. According to the initial laboratory results, the early-progression group had significantly lower serum-albumin levels compared to the non-progression group. In addition, the number of patients with a high BUN (≥30 mg/dL) was greater in the early-progression group. The in-hospital mortality rate of patients with early progression to severe disease was higher than those without progression. The hospital length-of-stay also tended to be longer in the early-progression group (Table 1). Thirty-six patients died in hospital, with a median LOS of 11 days. No significant difference in initial median NEWS2 was observed in the non-survivors and the survivors.

### 3.2. Performance of NEWS2 in the Prediction of Early Progression to Severe Disease

The median NEWS2 of the early-progression group were higher than the non-progression group, with 8 (6–9) vs. 7 (5–8), *p* = 0.015, respectively. The hospital mortality of the patients with early-progression to severe pneumonia was significantly higher than that of the non-progression group (30.2% vs. 9.7%, *p* < 0.001, respectively). The median time of hospital-LOS was also longer in the early-progression group, at 15 vs. 10 days, *p* = 0.053 respectively (Table 1).

The characteristics of patients with CAP according to discharge status, comparing non-survivors vs. survivors, are shown in Table 2.

The AUROC of NEWS2 to predict early progression to severe disease was 0.61 (95% CI: 0.52–0.70). The best cut-off NEWS2 score was ≥ 8 (Table 3). The AUROC of NEWS2 ≥ 8, IDSA/ATS minor criteria ≥ 3, and CURB65 ≥ 3 were not significantly different, with 0.61 (95% CI: 0.52–0.70), 0.56 (95% CI 0.48–0.65), and 0.58 (95% CI 0.49–0.66), respectively (Table 4).

**Figure 1:**
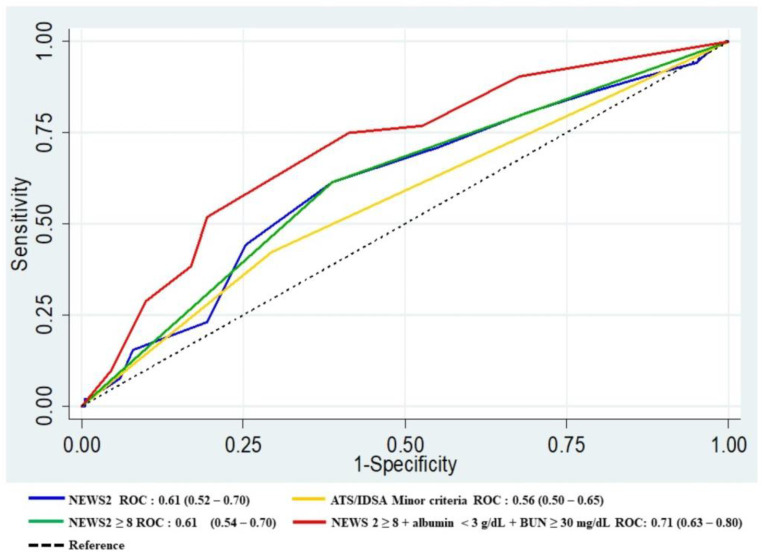
ROC curve for predicting early progression to severe CAP: comparisons between ATS/IDSA minor criteria, NEWS2, NEWS2 ≥ 8, and NEWS 2 ≥ 8 + Albumin < 3 g/dL + BUN ≥ 30 mg/dL.

**Figure 2:**
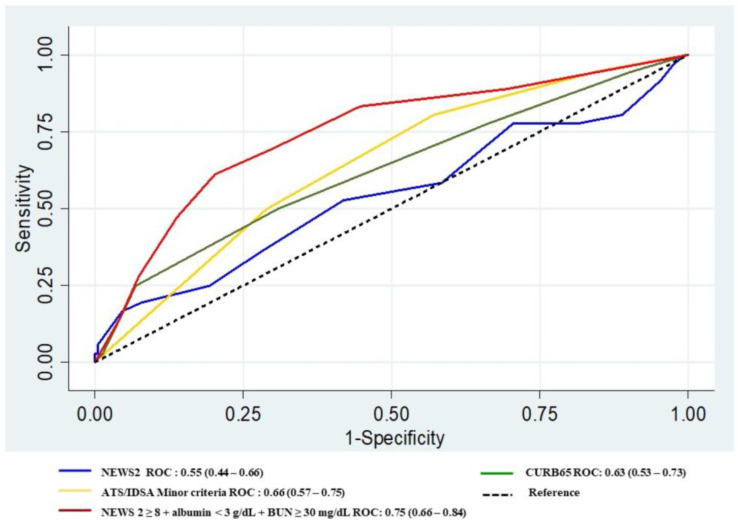
AUROC of NEWS2, IDSA/ATS minor criteria, and CURB65, to predict hospital mortality in patients with CAP.

The addition of albumin level < 3 g/dL and BUN ≥ 30 mg/dL to NEWS2 ≥ 8 improved the AUROC from 0.61 to 0.71, *p* = 0.015 (Table 4, Figure 1). The accuracy of NEWS2 to predict in-hospital mortality was lower than the IDSA/ATS, CURB65, and NEWS2 ≥ 8 in combination with albumin level < 3 g/dL and BUN ≥ 30 mg/dL, with AUROC of 0.55 vs. 0.66 vs. 0.63 vs. 0.75, respectively (Figure 2).

## 4. Discussion

This was the first prospective cohort study that compared the accuracy of NEWS2 to 2007 IDSA/ATS minor criteria and CURB65 in the prediction of early progression to severe CAP. When patients progress to severe CAP, they have significantly greater mortality and longer hospital length-of-stays than patients with non-severe CAP. Our findings show that IDSA/ATS 2007 minor criteria, as well as initial NEWS2 for early progression to severe CAP, had a low predictive performance. The cut-off of NEWS2 at ≥ 8 did not improve the result. The assessment of the commonly used scores including CURB-65 ≥ 3 also showed low predictive-performance, with AUROC < 0.7. However, significant improvement in the discriminative value was found after adding clinical parameters including low serum-albumin level and high BUN level. The best cut-off value of the BUN level to predict poor outcomes in this study was ≥ 30 mg/dL, while the BUN level used in the CURB-65 and the 2007 IDSA/ATS was ≥ 20 mg/dL. Our study was also found albumin level < 3 g/dL and BUN ≥ 30 mg/dL added to NEWS2 ≥ 8 increased the predictive value for mortality, shown in Figure 2.

Several studies demonstrated a favorable predictive-performance of IDSA/ATS minor criteria for ICU admission, the AUROC 0.85–0.88 [8,9,10]. Two systematic reviews and meta-analyses in 2011 and 2012 demonstrated moderate sensitivity (55.7–57.0%) and high specificity (90.5–91.7%) of IDSA/ATS minor criteria for prediction of ICU admission, while CURB65 provided lower sensitivity (50.0–56.2%) and specificity (72.1–74.2%) [5,6]. The AUROC of CURB65 was 0.69 [5]. Sbiti-Rohr et al. reported a low discrimination for NEWS in predicting thirty-day and six-year mortality in patients with CAP presenting at the Emergency Department, with AUROC < 0.70. However, it showed a moderate prediction for ICU admission within 30 days (AUROC 0.73) and additional predictive-benefit when added to the PSI and CURB65 [15]. According to the data of the current study, the predictive accuracy of the IDSA/ATS and the CURB65 for ICU admission and mortality were lower than previously reported. The differences in mortality rate and primary outcomes could be the explanation.

At the present, NEWS2 has been widely used in clinical practice, aiming at early detection and making appropriate responses for clinical deterioration in patients with acute illness. The results from this study confirm that the accuracy of initial NEWS2 to predict early progression to severe disease was comparable to the known tools, including IDSA/ATS and CURB-65. The additional advantage of NEWS2 over the other tools is that NEWS2 does not require laboratory results, especially the arterial-blood-gas test to measure the PaO_2_/FiO_2_ ratio, which is an invasive procedure and may not be available in all hospitals. NEWS2 can be quickly evaluated at the bedside by many levels of health-care workers, using the routine vital-signs measurement and the level-of-consciousness test. Thus, it can be inserted into the nurse-driven protocol to alert physicians and triage the level of care in patients with CAP, who present with a mild disease initially. The association between low albumin-levels (≤3.1 g/dL) and poor outcomes, including longer time of mechanical ventilation and vasopressors in sepsis patients, has been reported earlier [16]. Holder AL et al. demonstrated that serum albumin < 3.5 g/dL was independently associated with disease progression to severe sepsis or shock within 96 h [17]. Several studies demonstrated that high BUN-levels are associated with increased mortality in sepsis patients; however, the cut-off varies in each study [18,19,20]. Moreover, a high serum-BUN-to-albumin ratio was found to be a good predictor of mortality in patients with sepsis and septic shock [21,22,23]. This study found the addition of the high-BUN and low-albumin values to NEWS2 helped prediction of early progression to severe disease in patients with non-severe CAP. However, further external-validation studies are required to confirm our findings.

Our study did have some limitations. First, it was a single-center study that enrolled only CAP patients who presented at the ED and were admitted to hospital. We did not follow the patients who were discharged from the ED. There was a possibility that patients were subsequently admitted to another hospital, due to clinical deterioration. In addition, we did not collect subsequent NEWS2 after patient admission, which could be more useful in real-time clinical assessment than a single-score measurement. Further study aimed at evaluating the benefit of NEWS2 for the prediction of severe disease and/or mortality with CAP, in the setting of general and community hospitals is recommended. Lastly, the result of our study may not be applicable to COVID-19 pneumonia. Further study to evaluate the role of NEWS2 in the COVID-19 setting would be interesting.

## 5. Conclusions

The NEWS2 could help us to plan appropriate bed-management and effective use of limited resources. The study results support the routine use of screening for NEWS2 in non-COVID-19, CAP patients. The predictive accuracy for early progression to severe pneumonia of NEWS2 was comparable to 2007 IDSA/ATS minor criteria and CURB65. The cutoff of initial NEWS2 ≥ 8 is suggested. The combination of NEWS2 ≥ 8, albumin < 3 g/dL, and BUN ≥ 30 mg/dL was a good predictor of early progression to severe disease and mortality in patients with CAP.

## Figures and Tables

**Table 1:** Characteristics of patients with community-acquired pneumonia between those with early progression to severe pneumonia and those with non-progression within 72 h.

Characteristic	Early Progression (N = 53)	Non-Progression (N = 207)	*p*-Value
Age, years, mean ± SD	70.6 ± 15.4	70.9 ± 17.2	0.898
Male, n (%)	16 (30.2)	73 (35.3)	0.521
Comorbidities, n (%)			
- Hypertension	21 (39.6)	89 (43.0)	0.756
- Diabetes mellitus	12 (22.6)	48 (23.2)	1
- Cerebrovascular disease	11 (20.8)	46 (22.2)	1
- Coronary artery disease	9 (17.0)	22 (10.6)	0.234
- Chronic kidney disease	8 (15.1)	49 (23.7)	0.198
- Liver disease	2 (3.8)	6 (2.9)	0.667
- Chronic lung disease	6 (11.3)	35 (16.9)	0.401
- Autoimmune disease	3 (5.7)	7 (3.4)	0.431
- Malignancy	14 (26.4)	44 (21.3)	0.460
- HIV infection	1 (1.9)	7 (3.4)	1.000
Symptoms			
- Fever	30 (56.6)	136 (65.7)	0.262
- Productive cough	33 (62.3)	113 (54.6)	0.354
- Dyspnea	41 (77.4)	131 (63.3)	0.073
- Pleuritis chest pain	1 (1.9)	10 (4.8)	0.470
Signs			
- Crepitation	43 (81.1)	172 (83.1)	0.690
- Rhonchi	10 (18.9)	35 (16.9)	0.690
Lab			
Hb, g/dL, mean ± SD	10.7 ± 2.6	10.8 ± 3.0	0.781
WBC, cells/mm^3^, median (IQR)	12,140 (8560, 16,440)	11,040 (7350, 15,060)	0.226
% PMN, mean ± SD	82.3 ± 10.6	79.7 ± 13.0	0.173
% Lymphocyte, median (IQR)	8.2 (4.0, 14.8)	9.4 (6.2, 15.4)	0.103
N/L ratio, median (IQR)	10.4 (4.8, 23.4)	8.6 (4.9, 13.7)	0.075
Platelet, ×10^3^ cells/mm^3^, median (IQR)	234.0 (174.5, 316.5)	245.0 (163.0, 335.0)	0.668
BUN, median (IQR)	23.5 (16.0, 45.8)	20 (14, 32)	0.077
BUN ≥ 30, n (%)	24 (46.2)	59 (28.6)	0.02
Albumin, mean ± SD	3.0 ± 0.7	3.3 ± 0.6	0.005
Albumin < 3, n (%)	23 (44.2)	49 (24.9)	0.006
Lactate, median (IQR)	2.1 (1.5, 3.2)	2.0 (1.4, 2.7)	0.445
Chest X-ray, n (%)			
Multilobar infiltration	12 (22.6)	40 (19.3)	0.569
NEWS2, median (IQR)	8 (6, 9)	7 (5, 8)	0.015
NEWS2 ≥ 7, n (%)	37 (69.8)	112 (54.1)	0.044
IDSA/ATS minor criteria, n (%)			
RR ≥ 30	25 (47.2)	94 (45.4)	0.878
PaO_2_/FiO_2_ ratio ≤ 250	17 (32.1)	46 (22.2)	0.152
CXR: Multilobar infiltration	12 (22.6)	40 (19.3)	0.569
Confusion/disorientation	15 (28.3)	35 (16.9)	0.078
Uremia (BUN level ≥ 20 mg/dL)	32 (60.4)	97 (46.9)	0.091
Leukopenia (WBC < 4000 cells/µL)	1 (1.9)	6 (2.9)	1.000
Thrombocytopenia (Platelet count <100,000/µL)	5 (9.4)	12 (5.8)	0.353
Hypotension requiring aggressive fluid	12 (22.6)	32 (15.5)	0.222
resuscitation			
IDSA/ATS, n (%)			0.032
- 0	4 (7.5)	36 (17.4)	
- 1	8 (15.1)	57 (27.5)	
- 2	19 (35.8)	55 (26.6)	
- ≥3	22 (41.5)	59 (28.5)	
CURB65, median (IQR)	2 (2, 3)	2 (1, 3)	0.023
CURB65 ≥ 3, n (%)	24 (45.3)	62 (30.0)	0.049
Outcome			
Progress to respiratory failure required intubation, n (%)	42 (79.2)	23 (11.1)	<0.001
Progress to shock required vasopressor, n (%)	17 (32.1)	10 (4.8)	<0.001
Hospital LOS, median day (IQR)	15 (8, 22)	10 (6, 19)	0.053
In-hospital death, n (%)	16 (30.2)	20 (9.7)	<0.001

Abbreviations: Hb: hemoglobin, WBC: white blood count, IQR: interquartile range, PMN: Polymorphonuclear neutrophil, BUN: blood urea nitrogen, NEWS2: National Early Warning Score 2, IDSA/ATS: Infectious Diseases Society of America/American Thoracic Society, RR: respiratory rate, CURB65: confusion, uremia, respiratory rate, blood pressure, aged 65 or older, LOS: length of stay, SD: standard deviation.

**Table 2:** Characteristics of patients with community-acquired pneumonia of non-survivors and survivors groups.

Characteristic	Non-Survivors	Survivors	*p*-Value
	(N = 36)	(N = 224)	
Age, years, mean ± SD	74.7 ± 15.9	70.2 ± 16.9	0.135
Male, n (%)	25 (69.4)	146 (65.2)	0.617
Comorbidities, n (%)			
- Hypertension	13 (36.1)	97 (43.3)	0.417
- Diabetes mellitus	7 (19.4)	53 (53.7)	0.577
- Cerebrovascular disease	6 (16.7)	51 (22.8)	0.411
- Coronary artery disease	6 (16.7)	25 (11.2)	0.344
- Chronic kidney disease	5 (13.9)	52 (23.2)	0.209
- Liver disease	1 (2.8)	7 (3.1)	0.911
- Chronic lung disease	4 (11.1)	37 (16.5)	0.72
- Autoimmune disease	1(2.8)	9 (4.4)	0.001
- Malignancy	1.6 (44.4)	42 (18.8)	0.249
Symptoms, n (%)			
- Fever	22 (61.1)	144 (64.3)	0.938
- Productive cough	20 (55.6)	126 (56.3)	0.227
- Dyspnea	27 (75.0)	145 (64.7)	0.371
Signs			
- Crepitation	29 (80.6)	186 (83.0)	0.715
- Rhonchi	7 (19.4)	38 (17.0)	0.715
Lab			
Hb, g/dL, median (IQR)	10.1 ± 2.8	10.9 ± 2.9	0.161
WBC, cells/mm^3^, median (IQR)	12380 (8670, 15,920)	10740 (7447, 15,147)	0.291
% PMN, median (IQR)	84.0 ± 8.7	79.6 ± 13.0	0.054
% Lymphocyte, median (IQR)	8.2 (4.7, 11.2)	9.4 (5.3, 15.6)	0.056
N/L ratio, median (IQR)	10.4 (7.5, 17.2)	8.5 (4.7, 15.3)	0.061
Platelet, × 10^3^ cells/mm^3^, median (IQR)	245.0 (172.7, 370.0)	243 (164.5, 324.0)	0.486
BUN, median (IQR)	31.0 (16.5, 62.2)	20.0 (14.0, 32.2)	0.005
Albumin, median (IQR)	2.9 ± 0.6	3.3 ± 0.6	< 0.001
Lactate, median (IQR)	2.2 (1.5, 3.4)	1.9 (1.4,2.7)	0.417
Chest X-ray			
- Multilobar infiltration	9 (25.0)	43 (19.2)	0.419
NEWS2, median (IQR)	8 (6, 9.8)	7 (5, 9)	0.343
RR ≥ 30	16 (44.4)	103 (46.0)	0.864
PaO_2_/FiO_2_ ratio ≤ 250	9 (25.0)	43 (19.2)	0.419
Confusion/disorientation	13 (36.1)	37 (16.5)	0.006
Uremia (BUN level ≥ 20 mg/dL)	26 (72.2)	103 (46.0)	0.003
Leukopenia (WBC < 4000 cells/µL)	3 (8.3)	4 (1.8)	0.058
Thrombocytopenia (platelet count < 100,000/µL)	4 (11.1)	13(5.8)	0.232
Hypothermia (core temperature < 36 °C)	1 (2.8)	8(3.6)	0.809
Hypotension requiring aggressive fluid-resuscitation	9 (25.0)	35 (15.6)	0.164
IDSA/ATS, n (%)			0.023
- 0	2 (5.6)	38 (17.0)	
- 1	5 (13.9)	60 (26.8)	
- 2	11 (30.6)	63 (28.1)	
- ≥3	18 (50)	63 (28.1)	
CURB65, median (IQR)	2.5 (2, 3.7)	2 (1, 3)	0.009
CURB65 ≥ 3, n (%)	18 (50)	68 (30.4)	0.023
Outcome			
Hospital LOS, median day (IQR)	11 (4.5, 18.5)	11 (7.9, 19.0)	0.502

Abbreviations: SD: standard deviation, Hb: hemoglobin, WBC: white blood count, IQR: interquartile range, PMN: Polymorphonuclear neutrophil, BUN: blood urea nitrogen, NEWS2: National Early Warning Score 2, IDSA/ATS: Infectious Diseases Society of America/American Thoracic Society, RR: respiratory rate, CURB65: confusion, uremia, respiratory rate, blood pressure, aged 65 or over, LOS: length of stay.

**Table 3:** Sensitivity of NEWS2 at Initial Admission to Predict Early Progression of Pneumonia.

NEWS2 Score	Sensitivity (%)	Specificity (%)	LR +	LR −
≥6	79.2	31.9	1.16	0.65
≥7	69.8	45.9	1.29	0.66
≥8	60.4	62.3	1.60	0.64
≥9	43.4	75.4	1.76	0.75

**Table 4:** AUROC of NEWS2 when compared with IDSA/ATS minor criteria and other prediction tools to predict early progression of pneumonia.

	AUROC	95% CI	*p*-Value
NEWS2	0.61	0.52–0.70	Ref
IDSA/ATS minor ≥ 3	0.56	0.48–0.65	NS
CURB65 ≥ 3	0.580	0.49–0.66	NS
NEWS2 ≥ 8	0.61	0.54–0.70	NS
NEWS2 ≥ 8 + albumin < 3 g/dL + BUN ≥ 30 mg/dL	0.71	0.63–0.80	0.015

## Data Availability

The datasets that support the findings of this study are available on request from the corresponding author.
